# Supplementary material for: A classical chiral spin liquid from chiral interactions on the pyrochlore lattice
Source: Nat Commun. 2024 Nov 23;15:10162. doi: 10.1038/s41467-024-54558-7 (PMC11585553; doi:10.1038/s41467-024-54558-7)
Supplement: Supplementary file 1 — Supplementary Information [file 41467_2024_54558_MOESM1_ESM.pdf]

Supplemental Information for "A Classical Chiral Spin Liquid from Chiral Interactions on the Pyrochlore Lattice"

Daniel Lozano-Gómez,[1] Yasir Iqbal,[2] and Matthias Vojta[1]

[1]*Institut für Theoretische Physik and Würzburg-Dresden Cluster of Excellence ct.qmat, Technische Universität Dresden, 01062 Dresden, Germany*

[2]*Department of Physics and Quantum Centre of Excellence for Diamond and Emergent Materials (QuCenDiEM), Indian Institute of Technology Madras, Chennai 600036, India*

CONTENTS

Supplementary Note 1. Local z directions 2

Supplementary Note 2. Spin structure factor 2

Supplementary Note 3. Single-tetrahedron spin and gauge-field configurations 2

Supplementary Note 4. Color gauge fields and orthogonality of the fluxes 2

Supplementary Note 5. Vanishing single-tetrahedron magnetization 4

Supplementary Note 6. Effective Potts model 4

Supplementary Note 7. Thermodynamics from warm-up and cool-down schemes 5

Supplementary Note 8. Evolution of the nearest-neighbor spin correlations in the Heisenberg antiferromagnet 8

Supplementary Note 9. Finite-size effects 8

References 9

Supplementary Note 1. LOCAL z DIRECTIONS

In this section, we provide the local z direction of the spins in an up tetrahedron used in the definition of the emergent gauge fields $\mathbf{B}_\mu^{(c)}$. The local z directions $\mathbf{z}_\mu$ are given by the vectors

$$\mathbf{z}_0 = \frac{1}{\sqrt{3}}\left(111\right), \qquad \mathbf{z}_1 = \frac{1}{\sqrt{3}}\left(\bar{1}\bar{1}1\right), \tag{1}$$

$$\mathbf{z}_2 = \frac{1}{\sqrt{3}}\left(\bar{1}1\bar{1}\right), \qquad \mathbf{z}_3 = \frac{1}{\sqrt{3}}\left(1\bar{1}\bar{1}\right), \tag{2}$$

where μ labels the sublattice basis as shown in Fig. 1.

Supplementary Note 2. SPIN STRUCTURE FACTOR

In this section, we provide the expressions for the spin-spin correlation functions used in the main text. For the pyrochlore lattice, a non-Bravais lattice with a sublattice basis of 4, the most general correlation between the α and β components of the spins in sublattice μ and ν is given by

$$S_{\mu\nu}^{\alpha\beta}(\mathbf{k}) = \langle S_\mu^\alpha(\mathbf{k}) S_\nu^\beta(-\mathbf{k})\rangle. \tag{3}$$

Using this generic correlation function, the spin-spin correlation function is obtained by summing over the trace of spin components

$$\begin{aligned}\mathcal{S}(\mathbf{k}) &= \sum_{\alpha,\beta}\sum_{\mu,\nu}\delta_{\alpha\beta}S_{\mu\nu}^{\alpha\beta}(\mathbf{k}) \\ &= \sum_{\alpha,\beta}\sum_{\mu,\nu}\delta_{\alpha\beta}\langle S_\mu^\alpha(\mathbf{k}) S_\nu^\beta(-\mathbf{k})\rangle.\end{aligned} \tag{4}$$

For completeness, we provide the experimentally measurable unpolarized neutron structure factor,

$$\mathcal{S}_\perp(\mathbf{k}) = \sum_{\alpha,,\beta}\sum_{\mu,\nu}\left(\delta_{\alpha,\beta} - \hat{k}^\alpha\hat{k}^\beta\right)\langle S_\mu^\alpha(\mathbf{k}) S_\nu^\beta(-\mathbf{k})\rangle. \tag{5}$$

It is worth noting that, since the chiral Hamiltonian in Eq. (1) preserves a global O(3) symmetry in the spin degrees of freedom, the unpolarized neutron structure factor for this system presents the same features as those illustrated in the spin-spin correlation function with a diminished intensity originating from the neutron projection.

Supplementary Note 3. SINGLE-TETRAHEDRON SPIN AND GAUGE-FIELD CONFIGURATIONS

In this section, we provide the 12 single-tetrahedron spin $\mathbf{S}_\mu$, their corresponding gauge-field $\mathbf{B}_\mu^{(c)}$ configurations and the single tetrahedron flux $\mathbf{\Phi}^{(c)}$ of for the 12 distinct right-hand chiral single tetrahedron configurations, see Supplementary Fig. 1.

Supplementary Note 4. COLOR GAUGE FIELDS AND ORTHOGONALITY OF THE FLUXES

In this section, we provide further information regarding the three color gauge fields $\mathbf{B}_\mu^{(c)}$ and the associated total flux $\mathbf{\Phi}^{(c)} \equiv \sum_\mu \mathbf{B}_\mu^{(c)}$. Supplementary Figure 1(c) illustrates the 12 distinct right-hand chiral single tetrahedron configurations which can be used to tile the pyrochlore lattice producing configurations like the one illustrated in Supplementary Fig. 2 for a particular $\mathbf{k} = \mathbf{0}$ configuration.

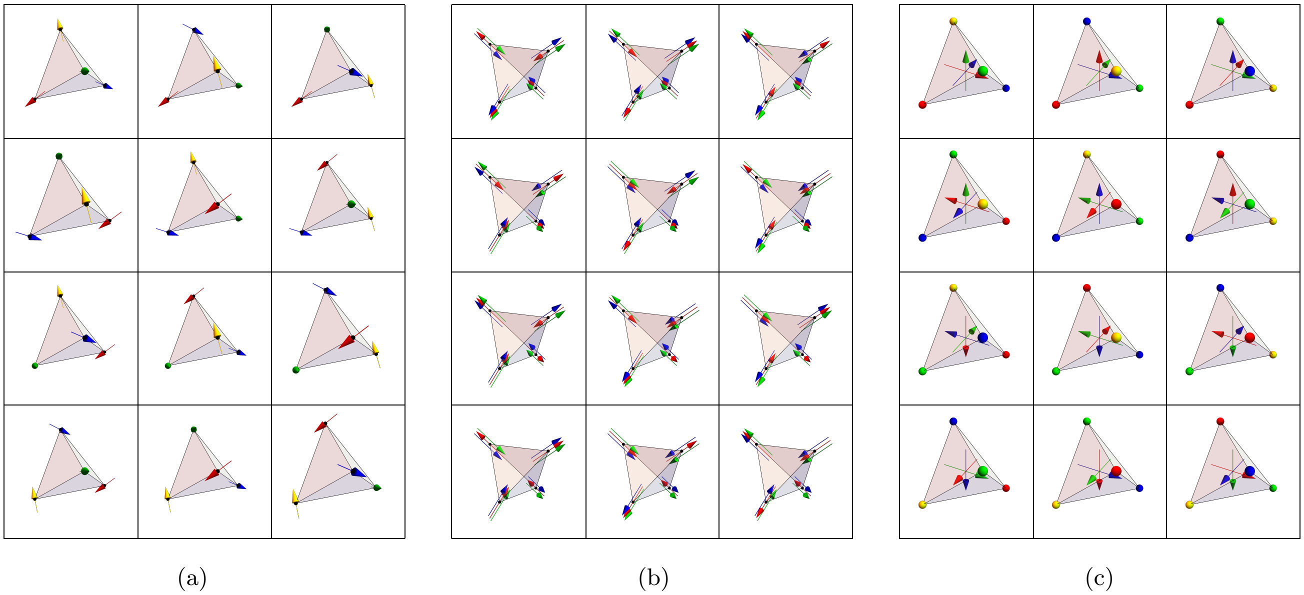

Supplementary Figure 1. (a) Single-tetrahedron spin $\mathbf{S}_\mu$ configurations where the spins in each tetrahedron are oriented along one of the $\{\mathbf{u}_0, \mathbf{u}_1, \mathbf{u}_2, \mathbf{u}_3\}$ orientations and colored according to the transformation $\{\mathbf{u}_0, \mathbf{u}_1, \mathbf{u}_2, \mathbf{u}_3\} \equiv \{R, B, G, Y\}$. (b) Single-tetrahedron gauge-field $\mathbf{B}_\mu^{(c)}$ configurations where the red, blue, and green colors correspond to the $\mathbf{B}_\mu^{(x)}$, $\mathbf{B}_\mu^{(y)}$ and $\mathbf{B}_\mu^{(z)}$ fields, respectively. (c) Single-tetrahedron ground-state configurations in the color basis with the total flux $\mathbf{\Phi}^{(c)}$ corresponding to the gauge fields $\mathbf{B}_\mu^{(c)}$ shown in the center of each tetrahedron where the red, blue, and green arrows correspond to the $\mathbf{\Phi}^{(x)}$, $\mathbf{\Phi}^{(y)}$, and $\mathbf{\Phi}^{(z)}$ fluxes, respectively.

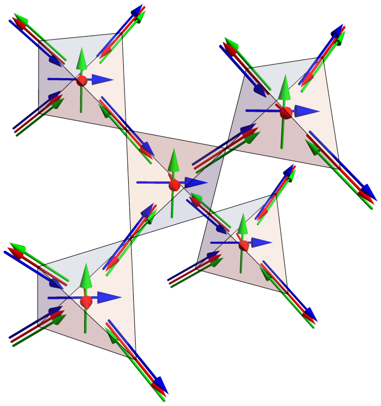

Supplementary Figure 2. Color gauge fields $\mathbf{B}_\mu^{(c)}$ defined on the pyrochlore lattice sites and the corresponding gauge flux $\mathbf{\Phi}^{(c)}$ through each tetrahedron shown in the center of the corresponding tetrahedra for a simple $\mathbf{k} = \mathbf{0}$ configuration.

As was previously pointed out in the main text, in the absence of gauge charges, the three color fluxes in every

single tetrahedron are constrained to be perpendicular. Indeed, from the definition of the color fields one obtains

$$\begin{aligned}\mathbf{\Phi}^b \cdot \mathbf{\Phi}^c &= \sum_\mu S^b_\mu \mathbf{z}_\mu \sum_\nu S^c_\nu \mathbf{z}_\nu \\ &= \sum_\mu S^b_\mu S^c_\mu - \frac{1}{3}\sum_{\mu\neq\nu} S^b_\mu S^c_\nu \\ &= \frac{16}{9}\delta_{b,c}, \end{aligned} \tag{6}$$

where we have used the fact that in the ground-state manifold $\sum_\mu S^c_\mu = 0$, and that distinct single-tetrahedron coloring configurations are orthogonal to each other.

Supplementary Note 5. VANISHING SINGLE-TETRAHEDRON MAGNETIZATION

In this section, we provide the distribution for all components of the single-tetrahedron magnetization obtained for the chiral Hamiltonian (1) at three distinct temperatures, see Supplementary Fig. 3. As discussed in the main text, all components of the single tetrahedron magnetization vanish as the temperature is decreased, implying that the ground state manifold is antiferromagnetic. This observation should, however, come as no surprise since a collinear configuration of spins in a single tetrahedron results in a high-energy configuration. This can be seen by simple inspection of the Hamiltonian in Eq. (1) as a collinear configuration results in a vanishing energy per tetrahedra.

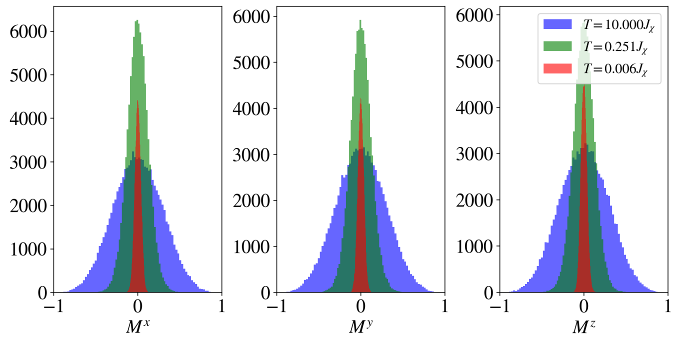

Supplementary Figure 3. Histogram of the single-tetrahedron magnetization components for three distinct temperatures of the chiral Hamiltonian in Eq. (1).

Supplementary Note 6. EFFECTIVE POTTS MODEL

In this section, we present the internal energy per spin, specific heat, and a snapshot of a low-temperature configuration for the chiral Potts model in Eq. (4) with $J = J_\chi = 1$ obtained via classical Monte-Carlo for both cool-down and warm-up schemes. In the cool-down simulations, the energy of the system displays a smooth evolution down to the lowest temperatures where it plateaus at a finite positive value, see the blue curve in Supplementary Fig. 4(a). On the other hand, the specific heat shows a Schottcky-like peak at a temperature of order proportional to the interaction parameters, i.e. $T \sim \mathcal{O}(J) = \mathcal{O}(J_\chi)$, see the blue curve in Supplementary Fig. 4(b). A similar peak in the specific heat is seen in spin-ice [1] as well as in the regular Potts model [2] where it signals the thermal depopulation of the magnetic monopoles and bionic charges, respectively. Although we could draw similar conclusions on the thermal depopulation of the bionic charges for this model, it is crucial to notice that states with no bionic charges are, by construction, those that have $E = 0$, implying that the low-temperature states sampled via Monte-Carlo have a non-vanishing population of bionic charges. Indeed, a closer inspection of the configurations sampled at temperatures below the Schottcky-like peak reveals that bions and left-hand chiral gauge field are present in the system, see Supplementary Fig. 4(c). As

discussed in the main text, these excitations become effectively immobile as the temperature is lowered which in turn gives them the characteristic behavior of fractons [3].

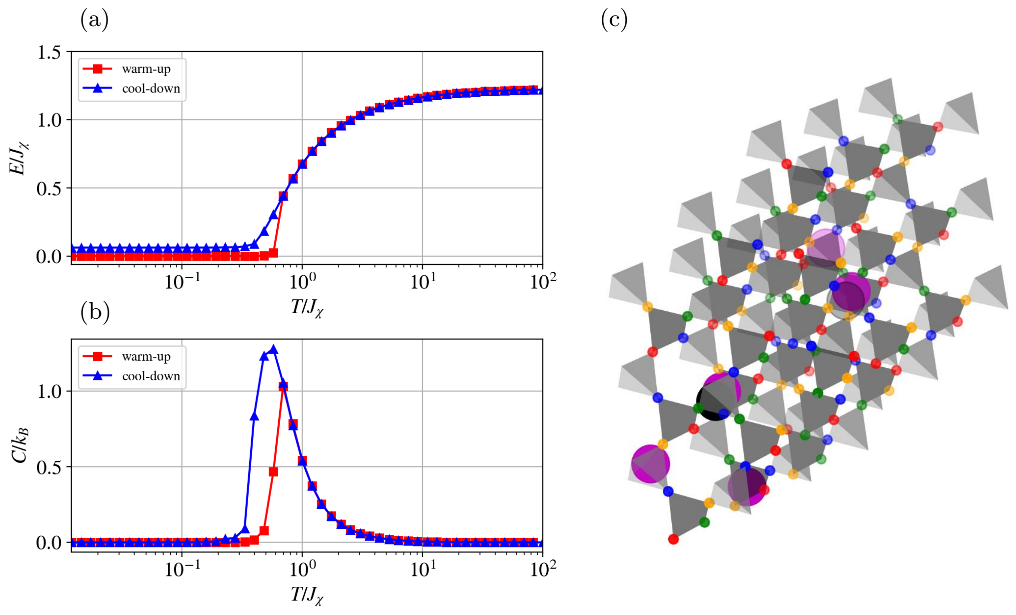

Supplementary Figure 4. (a) Internal energy and (b) specific heat obtained from classical Monte-Carlo for the chiral Potts model in Eq. (4) with $J = J_\chi = 1$. (c) Snapshot of a section of a classical Monte-Carlo simulation cell low-temperature configuration sampled via classical Monte-Carlo for the chiral Potts model in Eq. (4) with $J = J_\chi = 1$ and $J = 1$. Here the black (purple) circles mark the tetrahedra where the two-in-two-out (right-hand chiral) constraint is violated therefore leading to a high-energy configuration.

A similar situation is encountered when performing a warm-up simulation starting from a ground-state configuration, see red curve in Supplementary Fig. 4(a) and (b). In such a case the internal energy sampled by the classical Monte-Carlo does plateau at zero energy at low temperatures. However, and similar to the cool-down scheme, the warm-up scheme is frozen in the initial low-temperature configuration due to the high cost associated with generating and moving the fractonic charges.

Supplementary Note 7. THERMODYNAMICS FROM WARM-UP AND COOL-DOWN SCHEMES

In this section, we present the specific heat and energy evolution obtained through warm-up and cool-down cMC schemes used to study the chiral Hamiltonian in Eq. (1). In Supplementary Fig. 5, we show results for two initial configurations employed in the warm-up scheme, namely, (i) a $\mathbf{k} = \mathbf{0}$ configuration and (ii) a configuration obtained from a $\mathbf{k} = \mathbf{0}$ configuration by applying a non-local energy-conserving transformation on an infinite kagome plane. We henceforth refer to these initial states as the $\mathbf{k} = \mathbf{0}$ state and the kagome state, respectively. For the warm-up schemes, the energy and specific heat of both the $\mathbf{k} = \mathbf{0}$ and the kagome configurations result in a similar behavior where the internal energy plateaus to the predicted value of E_0, and a peak in the specific heat is observed. As discussed in the main text, the $\mathbf{k} = \mathbf{0}$ warm-up scheme presents a peak in the specific heat associated with the onset of the all-out order from which the warm-up system was initialized. The observation of this peak in the specific heat of the warm-up scheme is associated with the proliferation of charges which effectively melt the $\mathbf{k} = \mathbf{0}$ chiral order to a disorder paramagnet. A similar behavior is observed for the warm-up scheme obtained from the initial kagome state where the peak is then associated to the onset of a *partially* ordered configuration. Indeed, if we measure the mean sublattice magnetization [4]

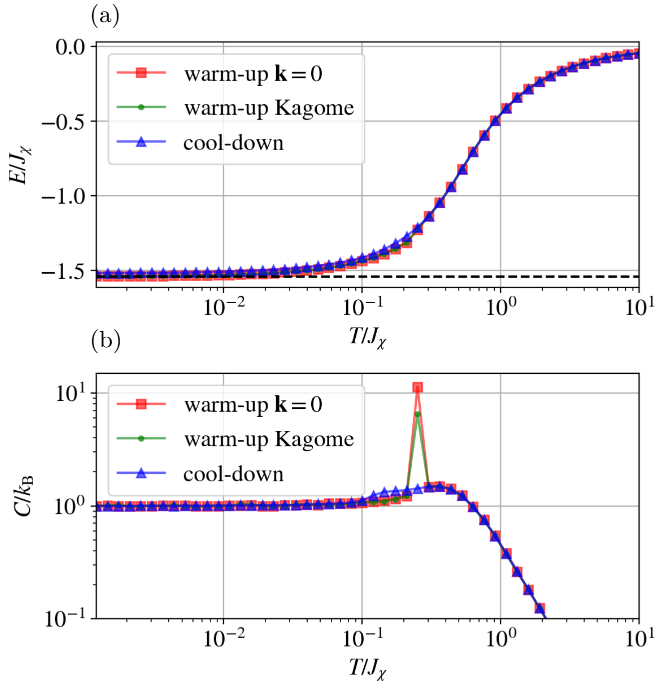

Supplementary Figure 5. (a) Internal energy and (b) specific heat per lattice site obtained using a warm-up and a cool-down scheme in a classical Monte-Carlo simulation. As in Fig. 6, the dashed line indicates the energy $E_0 = -1.5396 J_\chi$ predicted for a single-tetrahedron ground state.

$$m_s^2 = \frac{4}{N^2} \left\langle \sum_\mu \left| \sum_{\mathbf{r}} \mathbf{S}_\mu(\mathbf{r}) \right|^2 \right\rangle \tag{7}$$

where $\mathbf{r}$ labels the FCC positions, μ the sublattice index, and N corresponds to the number of spins in the system, we observe how this parameter saturates at one for the $\mathbf{k} = \mathbf{0}$ warm-up scheme and to an intermediate value for the kagome warm-up scheme, while it vanishes for the cool-down scheme, see Supplementary Fig. 6. In contrast, the observation of two bumps in the cool-down scheme is associated with two crossovers; one entering the antiferromagnetic manifold and a second one signaling the crossover to the chiral phase. The peak in the specific heat observed in Supplementary Fig. 5 in the warm-up simulations is associated with the freezing of the system into the initial partially ordered configuration. Indeed, our results suggest that the freezing observed in the warm-up scheme is bound to take place irrespective of the initial ground-state configuration selected.

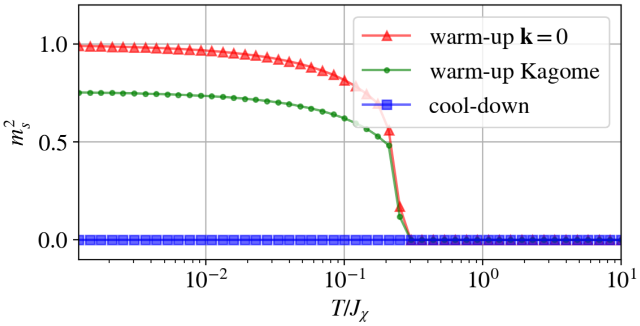

Supplementary Figure 6. Mean sublattice magnetization m_s^2 obtained using a warm-up and a cool-down scheme in a classical Monte-Carlo simulation.

Similar to the chiral Potts model, the discrepancy between the warm-up and cool-down schemes can be associated with the presence of non-vanishing charges. In the chiral Hamiltonian, however, the bionic and chiral charges are not quantized but are instead continuous degrees of freedom which can be thermally depopulated by spin fluctuations. A consequence of such a thermal depopulation is that the internal energy of the system sampled from a cool-down scheme slowly decreases with temperature, although it remains above that of the internal energy sampled with a warm-up scheme, as shown in Fig. 7 of the main text. Furthermore, the non-quantized character of the gauge charges for the chiral Hamiltonian refrains us from exactly locating their position in the lattice and producing an analysis similar to the one performed for the chiral Potts Hamiltonian in Supplementary Fig. 4 [5]. Instead, we can study the evolution of other statistical quantities which are directly associated with the ground-state manifold. Figure 6 in the main text and Supplementary Figure 7 illustrates the nearest-neighbor dot product and the single triangle chirality for warm-up and cool-down schemes sampled at different temperatures and averaged over 200 configurations, respectively. At low temperatures, all distributions are centered about the ground-state prediction (this being $-1/3$ for the nearest-neighbor dot product and $-4/(3\sqrt{3})$ for the chiral χ_{ijk} term). However, the distributions for the cool-down scheme are consistently broader than those of the warm-up schemes. This difference is associated with non-vanishing gauge charge excitations in the system.

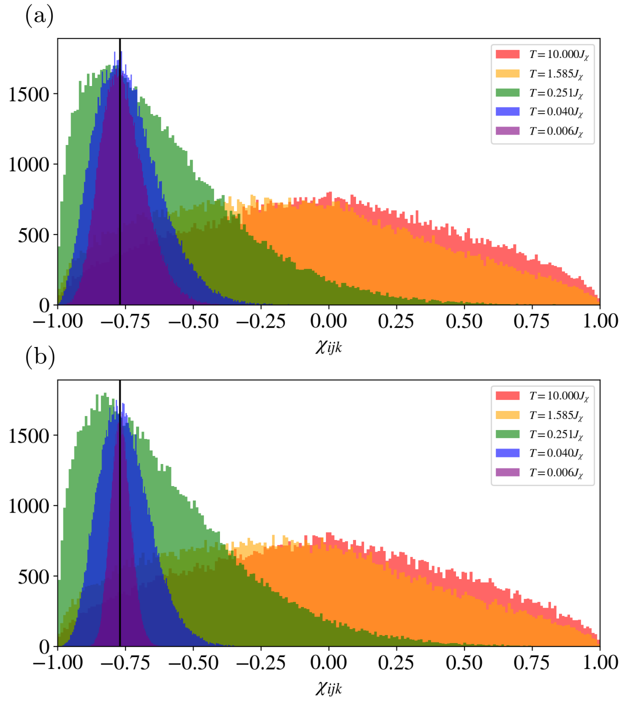

Supplementary Figure 7. Histogram of the chirality χ_{ijk} as defined in the main text in all the triangular phases of the pyrochlore tetrahedra, for distinct configurations sampled from our cMC simulations for different temperatures obtained for (a) a cool-down and (b) a warm-up scheme. Here, the vertical lines mark the predicted chirality for a single triangle in the ground-state manifold.

Lastly, we restate that a further study of both the chiral Hamiltonian in Eq. (1) and the chiral Potts Hamiltonian in Eq. (4) necessitates the implementation of a non-local update [6] capable of avoiding the freezing observed in the simulations and characterized in the main text. However, we emphasize that the analysis performed in this work already characterizes the intricate and rich physics which are observed in this system.

Supplementary Note 8. EVOLUTION OF THE NEAREST-NEIGHBOR SPIN CORRELATIONS IN THE HEISENBERG ANTIFERROMAGNET

In this section, we provide the evolution of the distribution of the dot product between nearest-neighbor spins for the pure Heisenberg antiferromagnetic model, see Supplementary Fig. 8. Although this distribution is not centered at $(-1/3)$, its average value is $(-1/3)$ which coincides with the average value one would obtain for the distribution in the chiral manifold. In fact, this equal average value motivated us to study the full distributions of the dot product instead of only its average to detect the onset of the chiral constraint as a function of temperature.

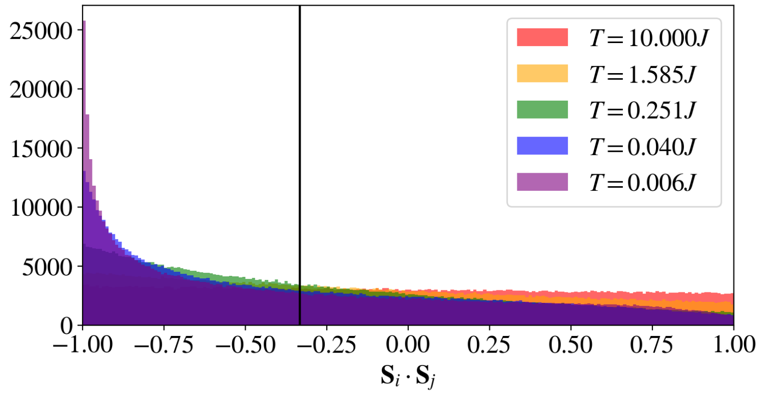

Supplementary Figure 8. Histogram of the nearest-neighbor spin correlations for different temperatures obtained for a cool-down scheme for the bare Heisenberg antiferromagnetic Hamiltonian.

Supplementary Note 9. FINITE-SIZE EFFECTS

To ensure that the analysis that we have presented is valid in the thermodynamic limit, we have performed a cMC simulations of the chiral Hamiltonian in Eq. (1) for three distinct system sizes with $N = 4L^3$ spins, namely $L = 6, 8, 10$. The specific heat thus obtained is illustrated in Supplementary Fig. 9 where the double-bump feature in the specific heat is preserved for all the system sizes studied. The agreement of the specific heat between these three system sizes suggests that the classical chiral spin liquid is indeed the phase realized by the chiral Hamiltonian in the thermodynamic limit at low temperatures.

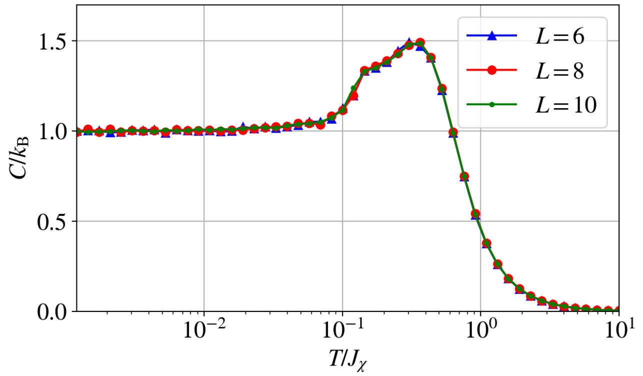

Supplementary Figure 9. Specific heat for the chiral Hamiltonian in Eq. (1) sampled through classical Monte-Carlo for three system sizes, namely $L = 6, 8, 10$.

[1] R. G. Melko and M. J. P. Gingras, Monte Carlo studies of the dipolar spin ice model, Journal of Physics: Condensed Matter **16**, R1277 (2004).

[2] V. Khemani, R. Moessner, S. A. Parameswaran, and S. L. Sondhi, Bionic Coulomb phase on the pyrochlore lattice, Phys. Rev. B **86**, 054411 (2012).

[3] R. M. Nandkishore and M. Hermele, Fractons, Annu. Rev. Condens. Matter Phys. **10**, 295 (2019).

[4] J. N. Reimers, Absence of long-range order in a three-dimensional geometrically frustrated antiferromagnet, Phys. Rev. B **45**, 7287 (1992).

[5] R. Flores-Calderón, O. Benton, and R. Moessner, Irrational Moments and Signatures of Higher-Rank Gauge Theories in Diluted Classical Spin Liquids, Phys. Rev. Lett. **133**, 106501 (2024).

[6] B. Placke, O. Benton, and R. Moessner, Ising fracton spin liquid on the honeycomb lattice, Phys. Rev. B **110**, L020401 (2024).
